# The Design and Positioning Method of a Flexible Zoom Artificial Compound Eye

**DOI:** 10.3390/mi9070319

**Published:** 2018-06-25

**Authors:** Lun Li, Yongping Hao, Jiulong Xu, Fengli Liu, Jiang Lu

**Affiliations:** 1 Department of Equipment Engineering, Shenyang Ligong University, Shenyang 110159, China; allen.li8910@gmail.com (L.L.); xjlsyit@gmil.com (J.X.); 2 Technology Center of Computer-aided Design & Manufacturing (CAD/CAM), Shenyang Ligong University, Shenyang 110159, China; fliu110159@gmail.com; 3 Department of Mechanical Engineering, Shenyang Ligong University, Shenyang 110159, China; 4 Fuyao Glass Industry Group Co., Ltd., Shenyang 110159, China; jianglu51769@gmail.com

**Keywords:** compound eye, zoom, polydimethylsiloxane (PDMS), aspherical surface, target positioning

## Abstract

The focal lengths of the sub-eyes in a single-layer uniform curved compound eye are all the same, resulting in poor imaging quality for the compound eye. A non-uniform curved compound eye can effectively solve the problem of poor edge-imaging quality, however, it suffers from a large spherical aberration, and is unable to achieve zoom imaging. To solve these problems, a new type of aspherical artificial compound eye structure with variable focal length is proposed in this paper. The structure divides the surface compound eye into three fan-shaped areas with different focal lengths of the microlens in different areas, which allow the artificial compound eye to zoom in a certain range. The focal length and size of the microlens is determined by the area and the location of the microlens. The aspherical optimization of the microlens is calculated, and spherical aberration in each area is reduced to one percent of the initial value. Through simulation analysis, the designed artificial compound eye structure realizes focal length adjustment and effectively reduces the problem of the poor imaging quality of the curved compound eye edge. As a result, an aspherical artificial compound eye sample—where the number of sub-eyes is *n* = 61, and the diameter of the base is Φ = 8.66 mm—was prepared by using a molding method. Additionally, the mutual relationship between the eyes of the child was calibrated, and hence, a mathematical model for the simultaneous identification of multiple sub-eyes was established. This study set up an experimental artificial compound eye positioning system, and through a number of microlens capture target point settlement coordinates, achieved an error value of less than 10%.

## 1. Introduction

Artificial compound eyes that are useful for application, are mostly of the planar structure type, which largely loses the advantage of insect compound eye structure: a large viewing angle [1]. Although many different types of curved compound eye preparation methods have been proposed, these methods have disadvantages, such as expensive processing equipment and complicated preparation processes, and hence, they still remain in the experimental stage [2]. There are many difficulties in the research of curved compound eyes, especially the structural design of curved compound eye imaging systems and the processing technology for curved microlens arrays [3,4,5]. 

In order to realize the characteristics of biological compound eyes and improve the imaging quality of the edge field of view, scholars have conducted many studies on bionic compound eyes [6,7,8,9,10]. Jeong et al. developed a bionic compound eye equivalent to compound insect eye dimensions [11]; the eye contained a total of 8370 sub-eyes with a maximum diameter of only 25 μm, and was densely arranged on the surface of a hemispherical polymer substrate with a diameter of 2.5 mm. However, this compound eye cannot be used for actual imaging. Radtke et al. [5] used laser lithography to fabricate a spherical bionic compound eye, consisting mainly of a microlens array distributed on a concave lens, and an array of apertures distributed on a convex lens, from which a distinguishable image was taken. In theory, this eye could contain 112 × 112 optical channels, covering a 31° × 31° field of view. However, due to approaching light optical channel crosstalk, which causes image ghosting, the available optical channels are only 40 × 40, and the corresponding field of view is only 10.3° × 10.3°. Li and Yi [12] have developed a high-precision surface microlens array with a total of 340 microlenses. The maximum profile error is only 5.6 microns, and the focal length is about 3.5 mm [13]. Floreano et al. developed a curved biomimetic compound eye, curved artificial compound eye (CurvACE), consisting of an optical layer, a photodetector array, and a flexible circuit board. The three components are accurately stacked, and the optical layer and photodetector array are cut line by line, ensuring the integrity of the flexible circuit board. Eventually, the entire setup is bent into a curved surface [14,15]. Song and others adopted an elastic composite optical element and a deformable silicon photodetector array to program a compound eye from a planar structure to effectively eliminate the off-axis aberration caused by the non-vertical axis of the microlens and image sensor [16]. A planer elastomeric microlens array that can be mechanically stretched to a very large extent was presented by Zhengwei et al. [17]. Li and Yi [18], using an ultraprecision diamond machining process, fabricated a microprism and ommatidia array on a curved and flat surface, respectively. An electrostatic deformation method is used to fabricate ommatidia arrays with different structures by changing the mask shape. However, the process is complicated, and the metal wire is likely to experience burnout when the voltage is too high. Moreover, the thickness of the polydimethylsiloxane (PDMS) is difficult to control in the spin process, and the cost is high. Although biomimetic structures that replicate insect compound eyes can be fabricated by these methods, these techniques require expensive facilities, and involve long processing times and complicated fabrication procedures. 

The spherical surface structure of the sub-apertures in compound eyes on a single-layer non-uniform curved surface leads to poor imaging quality of the compound eye, and cannot achieve zoom imaging [19]. In this paper, a new type of aspherical artificial compound eye structure with variable focal length is proposed. The structure divides the curved surface of the compound eye into several different imaging regions. The focal lengths of the sub-eyes in different imaging regions are different, so that the artificial compound eye can achieve focus adjustment within a certain range. The aspherical lens is optimized by Zemax (Zemax LLC, Kirkland, WA, USA) to reduce the spherical aberration of the microlens. Additionally, the compound eye model is traced by ray tracing to verify its imaging performance. A multiview positioning algorithm is proposed. By calibrating the position of each sub-eye in the three-dimensional model of the compound eye, the three-dimensional coordinates of a target point are obtained by solving the resulting over-determined equations using a least-squares method. The artificial compound eye was prepared by a molding process [20,21], and a compound eye imaging and positioning experiment was set up to test the imaging performance and target accuracy of the system.

## 2. Optimized Design of an Artificial Compound Eye: Aspherical Surface with Zoom 

**Figure 1:**
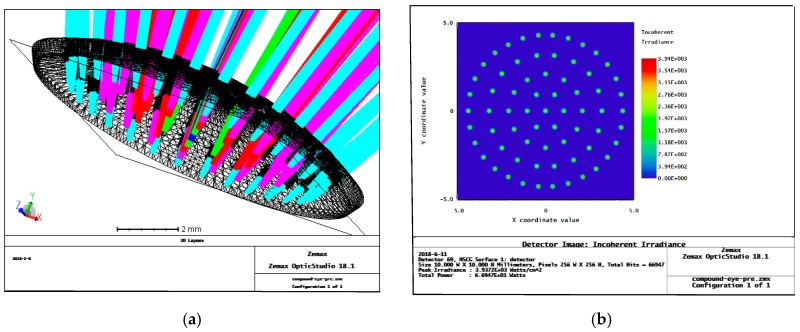
Non-uniform surface compound eye Zemax simulation trace results before optimization: (**a**) curved compound eye ray tracing; and different levels of sub-eyes use different colors of light to track; (**b**) detector energy pattern, the energy speckle of each sub-eyes is relatively divergent, and the peak irradiance is only 3.9372 × 10^3^ Watts/cm^2^.

With the continuous improvement of processing technology, research on insect compound eyes has made great progress [22,23]. By using an uneven surface in a compound eye, the problem of inaccuracy in each sub-eye is effectively solved. The Zemax ray tracing of a single-surface complex eye model is shown in Figure 1. According to the trace results, the incident ray corresponding to each sub-eye can be focused on a light detection target surface. However, due to the spherical structure of the surface of the microlenses at all levels, a large spherical aberration exists in the compound eye, which affects the overall imaging effects and has a great impact on target tracking and multi-target positioning in later uses of the compound eye [24]. Therefore, we performed aspheric optimization of the microlenses at all levels to improve the overall imaging quality of the compound eye.

### 2.1. Structural Design of the Variable Focal Length Artificial Compound Eye

**Figure 2:**
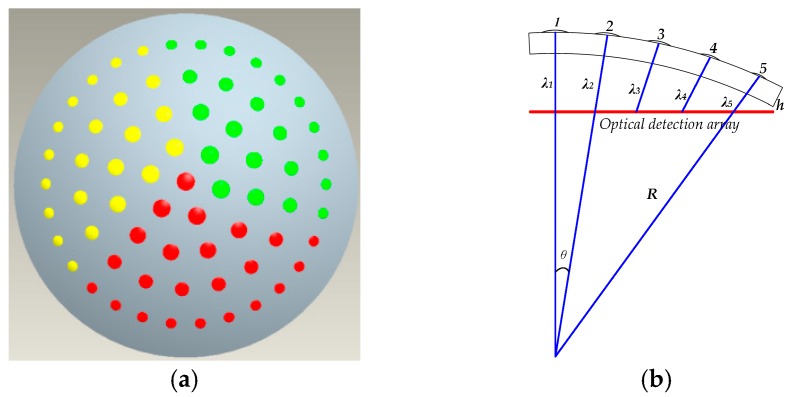
(**a**) Area division of the variable focus surface compound eye, where the three different colors represent the three regions divided; (**b**) the positional relationship between sub-eyes and the focal planes at all levels: *R*-curved base radius; *θ-*suborbital deflection angle; *λ*_1–5_-distance between the sub-eyes and the optical detection array.

The overall structure of the curved compound eye for zoom, as shown in Figure 2a, divides the curved surface into three equal parts, each with an angle of 120° and a corresponding “focal length” that is different. For the red, yellow, and green parts of the design, as depicted in Figure 2a, the focal lengths of the central small eye are 2.227 mm, 1.927 mm, and 2.527 mm, respectively. In this way, zooming can be achieved within a certain range to enhance the imaging performance of the artificial compound eye.

The microlens array on the curved substrate ultimately needs to be imaged on a planar photodetector array, which results in different distances from the center of microlenses that are at different locations on the substrate to the photodetection array. According to the geometric imaging principle, the effective focal length of the microlens is equal to the distance from the center of the microlens to the photodetection array, so only microlenses at the same position in each region have the same size. Apart from a central lens in the red area of the structure, the microlens arrays in the three areas have the same relative positions and numbers of lenses (Figure 2a).

The design parameters of the variable-focus surface compound eye structure are shown in Table 1.

The distance from the optical center of the central eye to the target surface of the photodetector is taken as a design reference, and the distance from the center of each microlens to the photodetection array is the effective focal length of each sub-eye [12,13]. Taking the sub-eye array in the red area (Figure 2a) as an example, as shown in Figure 2b, the sub-eyes are divided into five levels according to their deflection angle relative to the central sub-eye. The sub-eyes in each level also have the same distance from the target surface of the photodetector. Therefore, the focal length and radius of curvature are also the same for all sub-eyes in a level.

According to the geometric relationship between the radius of curvature of the lens and the deflection angle of the sub-eyes, the effective focal length of the sub-eyes in each level can be calculated. The distance *λ_n_* from the inner surface of the substrate corresponding to the center of the *n*th (*n* ≤ 5) sub-eye to the photodetector target surface is

(1) $$\lambda_{n}=R-\frac{R-\left(\lambda_{1}+h\right)}{\cos\left(n\theta \right)},n=2,3,4,5$$

*R—*curved base radius; *θ—*suborbital deflection angle; *λ*_1–5_—the distance between the sub-eye and the optical detection array.

The effective focal length of the sub-eyes at all levels can be obtained from the thin lens manufacturing equation

(2) $$\frac{1}{f_{n}}=\left(n_{i}-1\right)\left(\frac{1}{r_{n}}-\frac{1}{R}\right)$$

where *f_n_* is the effective focal length of the *n*th sub-eye, *r_n_* is the radius of curvature of the *n*th sub-eye, *R* is the radius of the curved surface base, and *n_i_* is the refractive index of the material selected for making the compound eye. According to Equation (2), the initial values of the effective radii of the sub-eyes in the red region can be obtained, as shown in Table 2.

**Figure 3:**
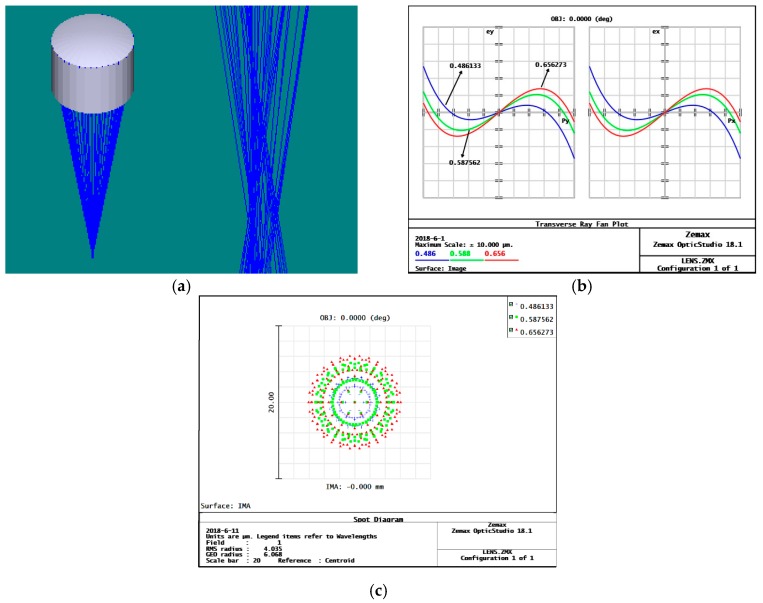
Spherical surface eye point simulation results: (**a**) spherical eye ray tracing; (**b**) spherical eye ray fan image, where p_x_, p_y_ is the entrance pupil coordinate; e_x_, e_y_ is the position of the light on the image plane, where the three different colors curves correspond to three different wavelengths of light; (**c**) spherical eye point diagram, where the three different colors point correspond to three different wavelengths of light.

According to the obtained initial parameters of each sub-eye, a parametric model of curved compound eye is established in Zemax, and ray tracing is performed to study the imaging performance of the sub-eyes of each level. By analyzing the ray tracing results of the first-degree sub-eye, Figure 3a, it can be seen that although the sub-eye can be imaged, the image does not converge to a single point. From the ray fan, Figure 3b, it is found that there is a large spherical aberration in the initial structure, and the ray aberration is 5.4 μm. From the dot plot, Figure 3c, it is seen that the radius of the Airy diffraction spot is 4.035 μm, which is relatively decentralized, and affects the imaging capability of the microlens.

In order to obtain the best imaging quality, aspherical optimization of the sub-eye structure is required. We establish a target optimization function in terms of the effective focal length (EFFL), spherical aberration (SPHA), and modulation transfer function (MIFT), setting optimization target values and weights for each. Then, to optimize the sub-eye’s spherical aberration, we determine the radius of curvature for each level that obtains the best imaging quality.

**Figure 4:**
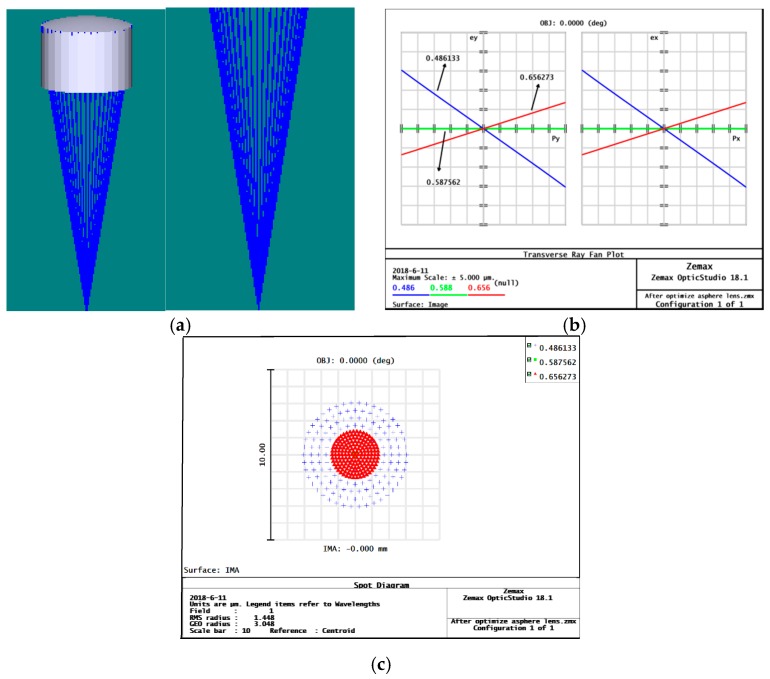
Aspherical sub-eye simulation results: (**a**) aspherical eye ray tracing; (**b**) aspherical surface light fan image, where p_x_, p_y_ is the entrance pupil coordinate, e_x_, e_y_ is the position of the light on the image plane, and the three different colors curves correspond to three different wavelengths of light; (**c**) aspherical face eyelet.

After optimization, the surface of the sub-eye becomes an aspherical structure, as shown in Figure 4. According to the Zemax ray tracing results, Figure 4a, the light is well focused to a point by the aspherical sub-eye, and no divergence of light occurs. At the same time, the analysis of the aspherical surface of the fan shows that the spherical aberration of the lens is greatly changed, and the aberration of the sub-areas are reduced to about 2.98 μm, as shown in Figure 4b. Observing the aspherical eyelet spot diagram, Figure 4c, the dispersion of the Airy diffraction spot has a root mean square radius of 1.448 μm and is relatively concentrated, which is beneficial to improving the imaging quality of the sub-eye lens.

The subocular surface is changed from a spherical surface to an aspheric surface, and the spherical aberration at each level of the sub-eye is reduced to one-hundredth of the initial structure. However, the non-spherical sub-eye structure puts forward higher requirements on the processing level of the compound mold for the later curved surface.

Table 3 shows the ball differences before and after optimization of the red zone subocular lens.

Table 4 shows the various dimensions of the optimized aspherical microlens in the red region. After the model is derived from Zemax, it is assembled to a corresponding position on the curved surface to form a three-focal-length microlens array.

### 2.2. Analysis of the Imaging Performance of the Zoomed Compound Eye Model after Optimization

**Figure 5:**
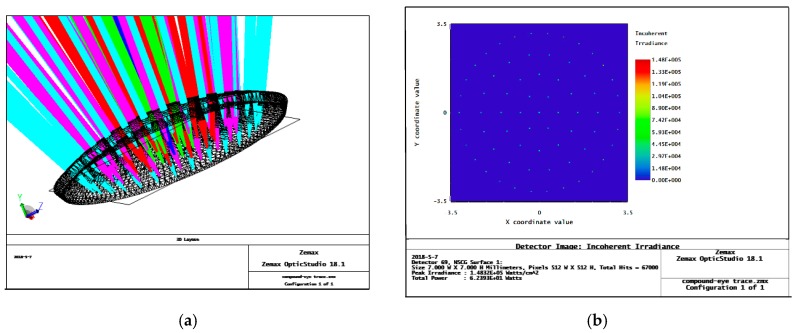
Ray tracing for the aspherical zoom surface compound eye model after optimization: (**a**) curved compound eye ray tracing, and different levels of sub-eyes use different colors of light to track; (**b**) the detector energy spot distribution; the energy speckle of each sub-eyes is relatively concentrated, and the peak irradiance is only 1.4832 × 10^5^ Watts/cm^2^.

After the optimization, a complex-eye surface model was established, and the 1–5 rays of the subocular microlenses in the surface model were traced using Zemax. The subocular lens arrays are arranged on a curved substrate and the lenses on each level have the same focal length. After optimization, the light focus and energy spot distribution of the sub-eyes at all levels are shown in Figure 5.

Comparing the results of the ray tracings in Figure 1 and Figure 5, we can find that before optimization, the size of the focused spot of each sub-eye is larger, and the light is scattered. Peak irradiance before optimization is only 3.9372 × 10^3^ watts/cm^2^. After optimization, the light levels of the sub-eyes of all levels are better focused, and have stronger spot energies on the detector, with a peak irradiance reaching 1.4832 × 10^5^ watts/cm^2^. This shows that the optimization of the light through our method is better; the imaging quality of the sub-eyes at all levels is improved, which provides a foundation for image recognition and positioning.

## 3. Multi-Eye Positioning Mathematical Model

According to the target imaging positioning mechanism, the target point, the center of the sub-eye lens, and the center of the target image point are collinear. If many sub-eyes on the compound eye can capture the target point and obtain a clear image at the same time, the location of the target point is at the intersection of the lines between the center of the target image and the corresponding sub-eye lens center. However, in actual imaging processing, due to the aberrations of the subocular lenses at all levels and the processing errors when preparing the curved surface of the compound eye, a non-linear distortion in the complex-eye image processing of the curved surface is unavoidable.

**Figure 6:**
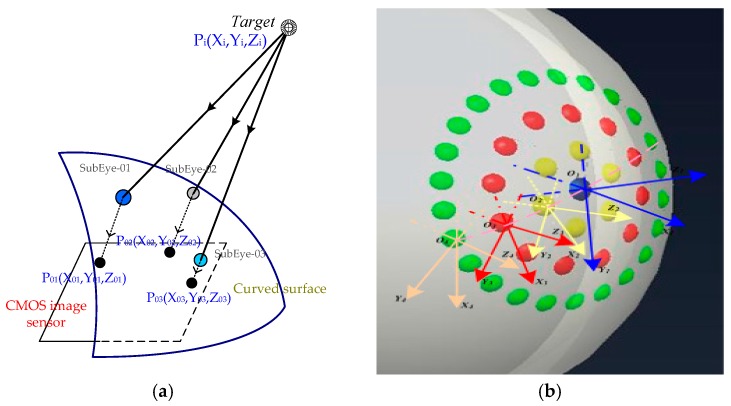
The principle and coordinate transformation of multi-eye positioning in artificial compound eyes: (**a**) the multi-eye positioning principle; and multiple sub-eyes can capture target image; (**b**) subordinate eye coordinate conversion diagram; the coordinate systems of all levels of sub-eyes are different, and coordinate transformation is needed for target positioning.

Due to the distortion, it is difficult to directly establish a mathematical correction model for object images. Thus, we divide the question into two parts: linear equations between the target point and subocular lens, and the correspondence between the subocular lens and the target image point. Schematics of these are shown in Figure 6a, using the two mathematical models established below to solve for the spatial coordinates of the target point.

As shown in Figure 6a, the sub-eye lens center coordinates are P_0*i*_ = (X_0*i*_, Y_0*i*_, Z_0*i*_), while the target point is P*_i_* = (X*_i_*, Y*_i_*, Z*_i_*). Thus, the direction vector ***p*** between the two points can be expressed as ***p*** *=* (tan*α_i_*, tan*β_i_*, 1), Let ***p***’(*a_i_, b_i_, c_i_*) = ***p***(tan*α*, tan*β*, 1), where *a* = tan*α*, *b* = tan*β*, *c* = 1, and the relationship between the subocular lens and the target point can be expressed as

(3) $$\frac{X_{i}-X_{0i}}{a_{i}}=\frac{Y_{i}-Y_{0i}}{b_{i}}=\frac{Z_{i}-Z_{0i}}{c_{i}}$$

where *i* = 1, 2, …, 61 is an index labelling each sub-eye lens.

For this calculation, the coordinate system of the sub-eye in the defined compound eye is the world coordinate system, and the deflection angle is between the 2–5-level sub-eye and the first-level sub-eye optical axis. In order to be unified in the calculation, it is necessary to unify the coordinate systems of the 2–5-level sub-eyes with the main coordinate system, as shown in Figure 6b. This coordinate system transformation follows the Euler transformation law. 

From the conversion relationship of Euler angles, we obtain the rotation matrix of the 2–5-level sub-eye coordinate systems to the main coordinate system:

$$M=\left[\begin{array}{ccc} \begin{array}{c} \cos\psi \\ \sin\psi \\ 0 \end{array} & \begin{array}{c} -\cos\theta \sin\psi \\ \cos\psi \cos\theta \\ \sin\theta \end{array} & \begin{array}{c} \sin\psi \sin\theta \\ -\sin\theta \cos\psi \\ \cos\theta \end{array} \end{array}\right]$$

where, ψ is the rotation angle between the 2–5 sub-eye coordinate system and the Z-axis, and θ is the rotation angle between the sub-eye coordinate system and the Y-axis.

Therefore, the relationship between the direction vector ***p***’ corresponding to each sub-eye in its local coordinate frame and the corresponding direction vector ***p*** in the world coordinate system is

***p*** = M***p***’. (4)

It is known that from the world coordinate of the lens center of each sub-eye, and the direction vector ***p*** in the world coordinate system of the corresponding sub-eye lens, the linear formula between the target point and the lens in the world coordinate system is obtained according to Equation (3):

(5) $$\left\{ \begin{array}{l} A_{i1}x+B_{i1}y+C_{i1}z+D_{i1}=0\\ A_{i2}x+B_{i2}y+C_{i2}z+D2=0 \end{array}\right.$$

If the target point can be captured by *n* sub-eyes at the same time, the relationship between the target point and the sub-eye lens can be expressed in terms in matrix notation:

RX = D(6)

where matrix R, and vectors X and D are

$$R=\left[\begin{array}{ccc} \begin{array}{c} A_{11}\\ \vdots \\ A_{n2} \end{array} & \begin{array}{c} B_{11}\\ \vdots \\ B_{n2} \end{array} & \begin{array}{c} C_{11}\\ \vdots \\ C_{n2} \end{array} \end{array}\right],X\;=\;{\left[x,\;y,\;z\right]}^{T}{,}_{\;}\;D\;=\;\left[-D_{11},\;-D_{12},\;\ldots ,\;-D_{n1},\;-D_{n2}\right].$$

To solve the over-determined equations, if R is full, let N = R^T^R, then the three-dimensional coordinates of the point X can be expressed as

X = N^−1^R^T^D (7)

## 4. Analysis on the Imaging Performance of a Zoom Compound Eye on a Curved Surface

**Figure 7:**
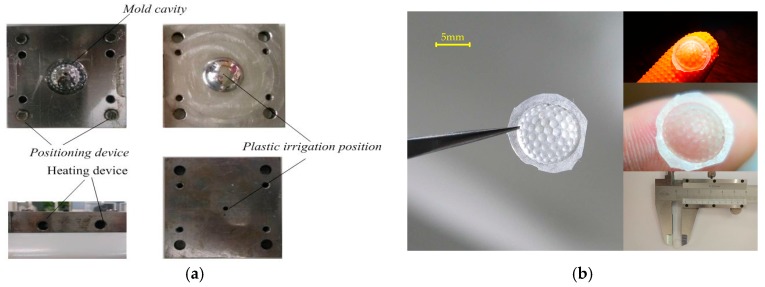
Preparation of curved compound eyes: (**a**) curved compound eye mold; (**b**) the finished product.

The method of mold forming is adopted in the preparation of a zoom curved-surface compound eye. This method has the advantages of simple operation, controllable precision, and batch preparation. The mold is made with a precision five-axis numerically controlled (NC) machine tool (Figure 7a) and polydimethylsiloxane (PDMS) as the forming material. The curing agent and PDMS were poured into the mold at a ratio of 1:10, and the temperature was fixed at 80 °C for one hour. After curing was completed, the compound eye sample was obtained, as shown in Figure 7b.

**Figure 8:**
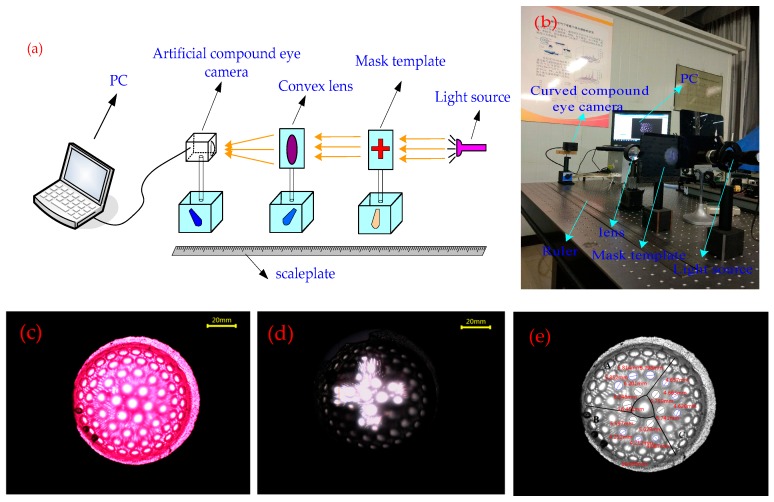
Zoom surface compound eye imaging experiment system and imaging analysis: (**a**) compound eye imaging experimental schematic; (**b**) photograph of the experiment setup; (**c**) curved compound eye collection on circular spot image; (**d**) curved compound eye collection on cruciform spot image; (**e**) sub-eye image size levels.

In order to verify the imaging performance of the prepared curved compound eye, an imaging test bench was constructed, as shown in Figure 8a,b. The experimental bench is mainly composed of a cluster light source (adjustable aperture size), a mask plate (circular, cross aperture), a convex lens, a curved compound eye camera, and a PC. By adjusting the distance between the components of the bench, the best imaging effect on the compound eye camera is obtained. Figure 8c shows the image collected by the compound eye camera when the light source was passed through a circular diaphragm. It can be seen from the image that the bright eyes of each sub-eye can be obtained at all levels. The image processing software ToupView is used to analyze the images collected in the compound eye. The three different color regions are denoted by A, B, and C, and the size of the eye spot in each region is measured. The imaging spot sizes in regions A, B, and C for each level are shown in Table 5.

Figure 8d shows when the light source passes through the cross-shaped diaphragm and the compound eye camera collects the image. In the area where the compound eye can receive light, multiple sub-eyes are imaged to form a “cross”, which is the same as the mask image. Due to the different focal lengths of the sub-eyes in the three imaging areas, the sizes of the sub-eye spots of the same grade in the “cross” image are different. By analyzing two different shapes of image information collected by the camera, it can be concluded that the designed zoom compound eye can achieve a certain range of zoom imaging.

## 5. Multi-Eye Positioning Experiment on the Artificial Compound Eye

**Figure 9:**
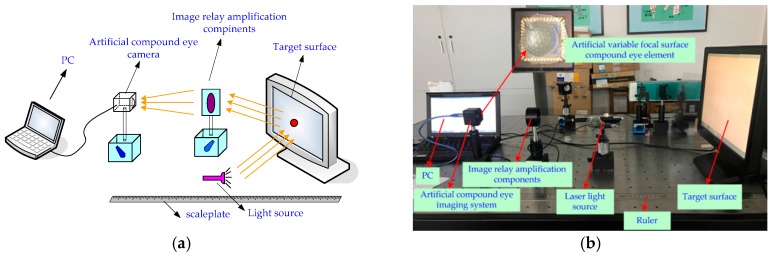
Multi-eye positioning experiment with an artificial compound eye: (**a**) schematic, and the light source illuminates the target surface to form a target image, and the compound eye camera captures the target image on the target surface; (**b**) photograph of test bench.

In order to perform a multi-eye positioning experiment, we employ a system consisting of an artificial compound eye, a complementary metal-oxide-semiconductor (CMOS) detector (CFV301-H2, DO3THINK, Shenzhen, China), a beam splitter, a laser light source, and a target surface, as shown in Figure 9. With the artificial eye and CMOS assembly, the focal length of the compound eye center is used as the regulation standard to adjust the distance between the surface of the artificial eye and the surface of the CMOS photodetector, so that all sub-eyes can obtain clear images. The CMOS optical detection array is used to receive multi-channel imaging of the target point and output the captured image to a PC for further processing.

Image acquisition was achieved by the CMOS photosensitive surface with a size of 6.55 mm × 4.92 mm, containing 2048 × 1536 effective pixels, each pixel having a unit size of 3.2 μm × 3.2 μm. The CMOS was connected through USB to a PC, whereupon the image could be processed further.

**Figure 10:**
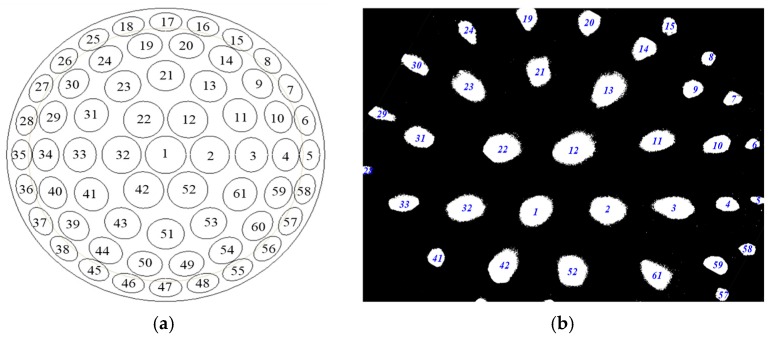
Multi-eye positioning experiment: (**a**) sub-eye lens number; and (**b**) sub-eye acquisition image.

In order to facilitate the determination of the coordinates of the sub-eye lenses during the target positioning process, each sub-eye lens needs to be numbered. The number of fly-eye lenses is shown in Figure 10a. According to the experimental conditions, to obtain the three-dimensional coordinates of the target, the following conditions need to be satisfied:

- The horizontal axis movement plane must be perpendicular to the target plane;
- The target plane must be parallel to the artificial eye compound plane;
- The intersection of the optical axis of the main compound lens with the target plane and the distance between the center of the main lens and the intersection point of the main compound lens must be the same.

The sub-eye imaging information collected by the experiment is shown in Figure 10b.

Through experimentation, the number of sub-eyes imaged at the target point was 33. In order to dissect the positioning error of the same target in the zoom-curved compound eye imaging system, calibration was performed. In the calibration process, the complex spherical eyeball center is used as the origin, and the target point coordinate is set to P_0_ = (18.6 mm, 12.52 mm, 85 mm). According to Equation (7), if the target point is captured by two or more sub-eye channels, its three-dimensional coordinates can be determined based on the composition of the over-determined equations. Therefore, the sub-eye channels 2–20 of the 20 lenses are extracted to solve for the three-dimensional coordinates of the spatial target point P_0_, as shown in Table 6.

**Figure 11:**
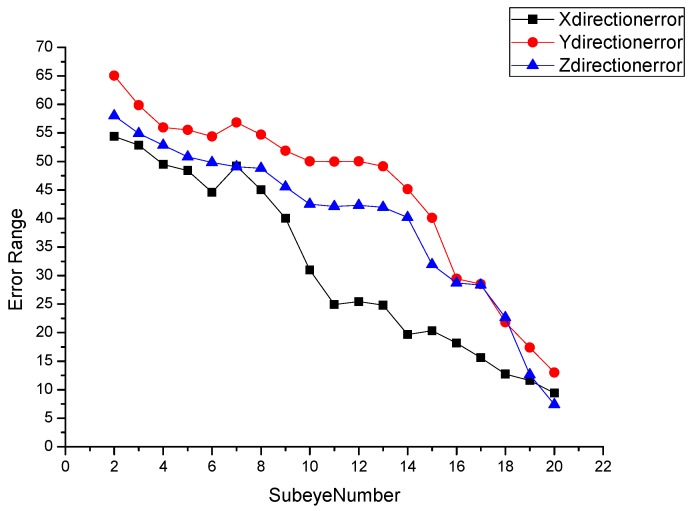
Error in the three-dimensional coordinates of the target point as a function of the number of captured sub-eyes.

From the coordinates of the target point shown in Table 6, the error in the calculated X, Y, and Z coordinate values of the target point are determined to gradually decrease as the number of captured target sub-eyes increases. The error curve in three directions is shown in Figure 11. Analysis of the trend of the curve can be obtained when the number of sub-eyes captured at the target point is less than 20, and the error of the coordinates of the calculated target point is larger. When the number of captured target sub-eyes reaches 20 or more, the error in the determined three-dimensional coordinates of the target object obtained by the curved compound eye positioning model is reduced to less than 10%.

Due to the errors in the preparation process of the compound eye on a curved surface, CMOS assembly error, measurement error of the target point, distance error between the target surface and the compound eye camera, and the like, the three-dimensional coordinates of the currently-solved target point have a relatively large error. These problems will be reduced as much as possible during subsequent experiments to improve the accuracy of the solution. Despite this, we have optimized the multi-eye positioning model, and proposed an improved method, such that the target positioning error is minimized.

## 6. Conclusions

In this paper, a non-uniform surface compound eye model with a variable focal length was proposed to solve the problem of the heterogeneous surface compound eye model. The design of the zoom compound eye model and optimization of the aspherical eye were studied. By subdividing the area of the surface model to achieve a certain range of zoom imaging, the sub-eye microlens was aspherically optimized to reduce spherical aberration on all levels of the sub-eye and improve the imaging quality of the surface compound eye. Focus was on the multi-eye positioning method for a curved surface compound eye. By establishing a multi-local positioning mathematical model, the incident angles of each channel corresponding to the three-dimensional target were inversely obtained, and the corresponding relationship between each subocular channel and its image point was established, thereby realizing the multi-object positioning of the curved surface. A non-uniform zoom compound eye was prepared, and the imaging effect of compound eye surface and the multi-local positioning algorithm were verified through experiments. The experimental results show that the greater the number of sub-eyes that captured the target point, the higher the positioning accuracy. When the number of captured sub-eyes exceeded 20, the fixed target positioning accuracy was within 10%.

## Figures and Tables

**Table 1:** Compound eye structure design parameters.

Base Radius	Angle between Adjacent Microlenses	Center to Edge Angle	Material	Refractive Index
4.5	6°	24°	PDMS ^1^	1.406

^1^ Polydimethylsiloxane (PDMS) is selected from SYLGARD 184 silicone elastomer produced by Dow Corning Corporation (Midland, MI, USA), the refractive index is 1.406 under natural light at room temperature (temperature: 25 °C; wavelength of light: 589.3 nm).

**Table 2:** Calculated initial parameters of subocular levels.

Sub-Eye Level	Level 1	Level 2	Level 3	Level 4	Level 5
*r_n_*(mm)	0.946	0.894	0.829	0.709	0.528
*f_n_*(mm)	2.227	2.173	2.009	1.724	1.302

**Table 3:** Ball differences for each level before and after sub-eye lens optimization.

Sub-Eye Level	Level 1	Level 2	Level 3	Level 4	Level 5
Pre-optimization (μm)	2.911	3.174	2.955	2.662	2.205
Post-optimization (μm)	0.023	0.020	0.019	0.067	0.037

**Table 4:** Aspherical sub-lens microlens size parameters.

Sub-Eye Level	Level 1	Level 2	Level 3	Level 4	Level 5
Focal length (mm)	2.227	2.173	2.009	1.724	1.302
Sub-eye surface radius of curvature (mm)	0.9298	0.9102	0.895	0.694	0.5149
Numerical aperture (mm)	0.638	0.624	0.59	0.501	0.382
Height (mm)	0.0561	0.0520	0.0489	0.0432	0.0345

**Table 5:** Sub-aperture spot size for each level in regions A, B, and C. Unit: mm.

Region	Level 2	Level 3	Level 4	Level 5
A	6.2235	5.821	5.251	4.885
B	5.613	4.184	3.905	3.685
C	5.75	4.646	4.297	4.306

**Table 6:** Calculated target point coordinate values and errors.

Number of Captured Sub-Eyes	Target Point Coordinates	X Error	Y Error	Z Error
2	(8.48130, 4.3735, 35.6823)	54.40%	65.06%	58.02%
3	(8.76780, 5.0243, 38.3295)	52.86%	59.86%	54.90%
4	(9.39870, 5.5143, 40.0674)	49.46%	55.95%	52.86%
5	(9.59450, 5.5663, 41.8220)	48.41%	55.54%	50.79%
6	(10.3022, 5.7101, 42.6291)	44.61%	54.39%	49.84%
7	(9.4464, 5.4071, 43.2847)	49.21%	56.81%	49.07%
8	(10.2208, 5.6696, 43.5180)	45.04%	54.71%	48.80%
9	(11.1493, 6.0254, 46.3066)	40.05%	51.87%	45.52%
10	(12.8394, 6.2577, 48.8626)	30.97%	50.01%	42.51%
11	(13.9621, 6.2632, 49.2046)	24.93%	49.97%	42.11%
12	(13.8682, 6.2564, 49.0375)	25.43%	50.02%	42.30%
13	(13.9867, 6.3666, 49.3266)	24.80%	49.15%	41.96%
14	(14.9411, 6.8690, 50.8268)	19.67%	45.13%	40.20%
15	(14.8166, 7.4969, 57.8406)	20.34%	40.12%	31.95%
16	(15.2169, 8.8299, 60.5823)	18.18%	29.47%	28.72%
17	(15.6933, 8.94870, 60.8983)	15.62%	28.52%	28.35%
18	(16.2307, 9.7869, 65.7329)	12.73%	21.82%	22.66%
19	(16.4409, 10.3442, 74.2530)	11.60%	17.37%	12.64%
20	(16.8539, 10.8893, 78.7162)	9.38%	13.02%	7.39%
